# Intimate partner violence during pregnancy and time to return to sexual activity after childbirth: analysis of the BRISA prenatal cohort

**DOI:** 10.1590/0102-311XEN094223

**Published:** 2024-06-17

**Authors:** Liendne Penha Abreu, Mônica Araújo Batalha, Liliana Yanet Gomez Aristizabal, Luciana Cavalcante Costa, Rosângela Fernandes Lucena Batista

**Affiliations:** 1 Universidade Federal do Maranhão, São Luís, Brasil.

**Keywords:** Violence Against Women, Intimate Partner Violence, Pregnancy, Sexual Behavior, Postpartum Period, Violencia Contra la Mujer, Violencia de Pareja, Embarazo, Conducta Sexual, Periodo Posparto, Violência Contra a Mulher, Violência por Parceiro Íntimo, Gravidez, Comportamento Sexual, Período Pós-parto

## Abstract

This study aimed to analyze whether there is an association between intimate partner violence during pregnancy and time to return to sexual activity after childbirth in the BRISA cohort in São Luís, Maranhão State, Brazil, between 2010 and 2013. This is a longitudinal study conducted with 665 women. Intimate partner violence during pregnancy was measured using an instrument created and validated by the World Health Organization to measure violence against women. Time to return to sexual activity after childbirth was investigated using a structured questionnaire. Logistic regression models were used to analyze whether there is an association between intimate partner violence during pregnancy and time to return to sexual activity after childbirth. The prevalence of violence by an intimate partner during pregnancy was 24.06%. The prevalence of women who returned to sexual activity within 3 months after childbirth was 67.96%. When analyzing the association between exposure and outcome, no association was found in the crude model (OR = 0.88; 95%CI: 0.60-1.30), nor in the adjusted model (OR = 1.00; 95%CI: 0.61-1.63). The study results highlight the importance of providing comprehensive care to women, considering both physical and psychological aspects, since violence has a significant impact on several aspects of women’s lives.

## Resumen

El objetivo de este estudio fue analizar si existe asociación entre la violencia por pareja íntima durante el embarazo y el tiempo para volver a la actividad sexual después del parto en la cohorte BRISA, en São Luis, Maranhão, Brasil, entre 2010 y 2013. Se trata de un estudio longitudinal realizado con 665 mujeres. Se midió la violencia por pareja íntima durante el embarazo a través de un instrumento para medir la violencia contra la mujer creado y validado por la Organización Mundial de la Salud. Se investigó el tiempo para volver a la actividad sexual después del parto a través de un cuestionario estructurado. Se utilizaron modelos de regresión logística para verificar si existe asociación entre la violencia por pareja íntima durante el embarazo y el tiempo para volver a la actividad sexual después del parto. La prevalencia de violencia perpetrada por pareja íntima durante el embarazo fue del 24,06%. La prevalencia de mujeres que volvieron a la actividad sexual dentro de los tres meses posteriores al parto fue del 67,96%. Al analizar la asociación entre la exposición y el resultado, se constató que no hubo asociación en el modelo crudo (OR = 0,88; IC95%: 0,60-1,30), ni en el modelo ajustado (OR = 1,00; IC95%: 0,61-1,63). Los resultados del estudio resaltan la importancia de proporcionar asistencia integral a la salud de la mujer, teniendo en cuenta tanto aspectos físicos como psicológicos, una vez que la violencia afecta significativamente varios aspectos de la vida femenina.

## Resumo

O objetivo deste estudo foi analisar se existe associação entre violência por parceiro íntimo na gestação e o tempo de retorno das atividades sexuais após o parto na coorte BRISA, em São Luís, Maranhão, Brasil, entre os anos de 2010 e 2013. Trata-se de estudo longitudinal conduzido com 665 mulheres. A violência por parceiro íntimo na gestação foi medida por meio de instrumento criado e validado pela Organização Mundial da Saúde para medir violência contra a mulher. O tempo de retorno das atividades sexuais após o parto foi investigado por meio de questionário estruturado. Modelos de regressão logística foram utilizados para verificar se existe associação entre violência por parceiro íntimo na gestação e tempo de retorno das atividades sexuais após o parto. A prevalência de violência perpetrada pelo parceiro íntimo na gestação foi de 24,06%. A prevalência de mulheres que retornaram às atividades sexuais em até três meses após o parto foi de 67,96%. Ao analisar a associação entre exposição e desfecho, observou-se que não houve associação no modelo bruto (OR = 0,88; IC95%: 0,60-1,30), nem no modelo ajustado (OR = 1,00; IC95%: 0,61-1,63). Os resultados do estudo evidenciam a importância de prestar assistência integral à saúde da mulher, considerando tanto aspectos físicos quanto psicológicos, uma vez que a violência tem impacto significativo em diversas áreas da vida feminina.

## Introduction

Violence against women is intrinsically linked with social, historical, and cultural aspects, reflecting the long history of gender inequality and oppression. Since ancient times, a patriarchal system has existed with undeniable submission to the dominant gender and in which men had economic, political, and sexual control over women. These gender norms, rooted in culture, often perpetuate violence, undermining women’s autonomy and well-being ^1^.

Intimate partner violence represents a serious public health problem and affects women all over the world ^2^. The phenomenon of violence against women can occur in different ways, affecting physical, property, psychological, moral and/or sexual aspects ^3^ in women of different ages, origins, ethnicities, social classes, marital statuses, education levels, and sexual orientations ^4^.

The pregnancy-puerperal cycle is a period in which more protection and care are expected for the mother-child binomial ^5^; however, national and international studies report a high prevalence of intimate partner violence during pregnancy ^6^ ^,^ ^7^ ^,^ ^8^ ^,^ ^9^ ^,^ ^10^ ^,^ ^11^. The occurrence of violence during this period or after birth is a reason for concern, as it can trigger obstetric complications that affect the health and quality of life of the mother and the fetus/neonate ^12^.

Pregnant women exposed to a situation of violence are more vulnerable to psychological suffering, with increased levels of stress, sadness, anguish, mental disorders, and suicidal ideation. It can lead to low adherence to prenatal care, risk of gynecological and obstetric problems such as urinary and vaginal tract infections, prenatal hospitalizations, serious maternal morbidities, and risk of miscarriage, intrauterine growth restriction, prematurity, perinatal death, and breastfeeding problems. In addition to physical and psychological consequences, it is believed that intimate partner violence may be related to sexuality after childbirth, but this relationship has not been fully explained in the literature ^13^.

Historically, women’s sexuality has often been ignored, discouraged or reduced to the role of reproduction, and during the postpartum period, this issue is even more neglected due to the focus on motherhood ^14^. The arrival of a child involves a number of emotional and social changes in the life of the woman, the family, and the parents, including hormonal, anatomical, psychological, and social changes, as well as different types of dissatisfaction regarding female health. In this period, up to 86% of women make sexual complaints, particularly in relation to dyspareunia and decreased sexual desire ^15^.

The main concerns expressed by women during this period include fear of pain, fear of a new pregnancy, baby care, and insecurity about their own bodies ^16^, in addition to anxiety and depression, particularly in the immediate postpartum period ^17^.

Although many women report a decline in sexual interest or desire after childbirth, about 80% of couples return to sexual activity within 12 week of childbirth. After six months, most women have resumed sexual intercourse and, after 12 months, most of them consider their sexual life similar to the pre-pregnancy period ^18^.

However, this period can vary, as several factors can influence sexual function and the return to sexual activity after childbirth, such as maternal age, breastfeeding, depression, tiredness, sexual inactivity in the first trimester of pregnancy, presence and degree of perineal injuries, body image after childbirth, concern about a new pregnancy, and urinary infection ^19^ ^,^ ^20^.

Sexuality has different meanings and expressions that are experienced by every woman in her daily life, making it an important aspect to be emphasized and referred to specialized care ^21^. Understanding how women experience sexuality after childbirth is required, since expectations for this period are not always similar, with distinct changes, contexts, and challenges ^22^.

In the context where women are exposed to violence, negotiations of sexual practices after childbirth may be more difficult, as they are subjected to the oppression of their violent partners. Also, a significant number of women do not receive information or guidance on sexual health during pregnancy, including when to return to sexual activity after childbirth ^23^. These women often return to sexual activity without desire, just to maintain intimacy and fulfill the expectations of their partners, which can contribute to the emergence of sexual health problems ^24^.

Although violence and sexual concerns are frequent problems in the pregnancy-puerperal cycle, studies on intimate partner violence and its relationship with women’s sexuality are still limited. There is a lack of studies that assess the physical and mental impacts of violence on women’s sexuality, particularly longitudinal studies. Therefore, our study aims to analyze whether there is an association between intimate partner violence during pregnancy and time to return to sexual activity after childbirth in the BRISA prenatal cohort, in the city of São Luís, Maranhão State, Brazil.

## Methods

### Study design

This is a longitudinal study conducted with data from the cohort for the study *Etiological Factors of Preterm Birth and Consequences of Perinatal Factors for Infant Health: Birth Cohorts in Two Brazilian Cities - BRISA*. The cohort began in 2010 and took place in São Luís and Ribeirão Preto (São Paulo State), with data collected in three stages: prenatal (baseline); 1st follow-up (at birth); and 2nd follow-up (between 12 and 35 months after childbirth). Our study used data from the three stages in the city of São Luís ^25^.

### Study site

São Luís is the capital of the state of Maranhão, the main city in the Greater São Luís Metropolitan Region. In 2010, the city had 1,014,837 inhabitants, with 375,093 women at childbearing age (10 to 49 years old), and an estimated population of 15,259 pregnant women ^26^.

### Participants and sample

A convenience sample was used in the prenatal phase. Pregnant women were contacted at a prenatal visit conducted up to the 5th month of pregnancy; they should have been submitted to at least one obstetric ultrasound exam before the 20th week of pregnancy, and have a single fetus and gestational age between 22 and 25 weeks at the time of data collection. In total, 1,447 pregnant women were interviewed from February 2010 to June 2011 ^27^.

Childbirths of pregnant women occurred from May 2010 to November 2011. A total of 1,381 mothers were interviewed once again within 24 hours after birth, constituting the childbirth sample.

**Figure 1:**
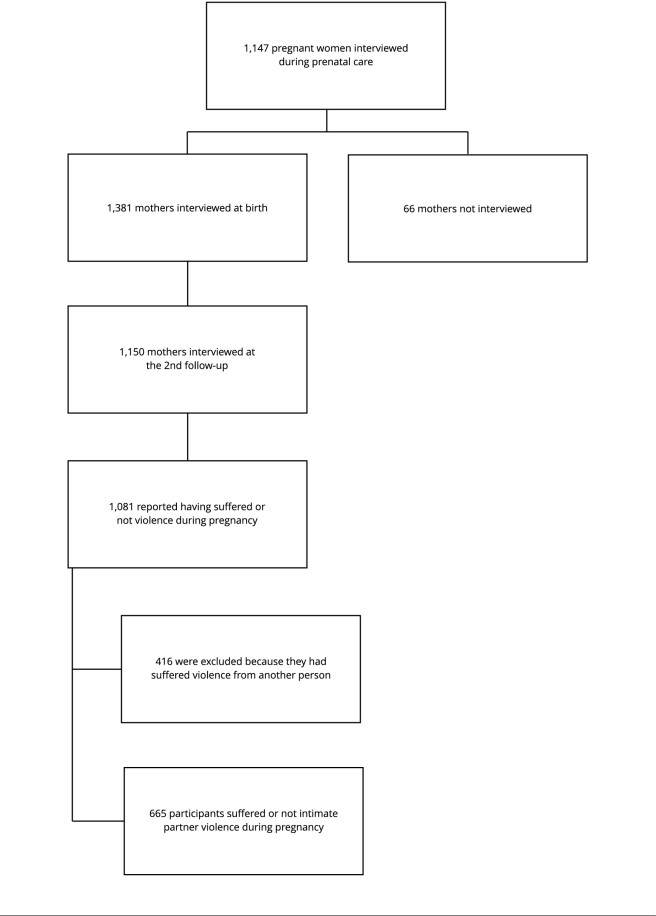
Flowchart of the population and sample of BRISA cohort participants analyzed in the study. São Luís, Maranhão State, Brazil.

Source: prepare by the authors.

The 2nd follow-up was conducted between September 2011 and March 2013. Participants were invited by telephone to attend the Maternal and Child University Hospital (HUMI, acronym in Portuguese), Federal University of Maranhão (UFMA, acronym in Portuguese), when the children were between 12 and 35 months old. In total, 1,151 mothers were interviewed; of those, 1,081 reported having suffered or not violence during pregnancy. For the purposes of this study, 416 interviewees who reported violence by another person were excluded, totaling 665 participants who did or did not suffer intimate partner violence during pregnancy (Figure 1).

### Data collection instruments

For data collection, interviews were conducted at three moments, with the application of structured questionnaires.

### Exposure variable

Violence during pregnancy was assessed using the *World Health Organization Violence Against Women* instrument (WHO VAW) ^28^, validated in Brazil ^29^, which has 32 self-administered questions, which investigates whether the woman has suffered violence during the current pregnancy and in the 12 months before that. The questionnaire has questions related to the occurrence and frequency of different types of violence (psychological, physical, and sexual violence).

In this study, exposure meant suffering intimate partner violence during pregnancy. This variable was measured through the question: “Who did this to you?” with the possible answers in the questionnaire: “current husband/partner/boyfriend”, “ex-husband/partner/boyfriend”, “father”, “stepfather”, “mother”, “stepmother”, “brother/sister/other family member who lives in the same house”, “family member who does not live with you”, “neighbor or other acquaintance”, “other”, and “there was no violence”. In this study, this variable was grouped into two categories: “those who did not suffer violence”, and “those who suffered intimate partner violence during pregnancy (current or former partner)”.

### Outcome variable

Time to return to sexual activity after childbirth was investigated using a questionnaire applied to mothers at the 2nd follow-up with the question: “When did you resume sexual activity after childbirth?”. The answers were: “0 to 14 days after birth”, “30 days after birth”, “1 to 3 months after birth”, “3 to 6 months after birth”, “9 months after birth”, “has not yet returned to sexual activity”, and “I don’t know”. However, in this study, we chose to categorize it as “up to 3 months after birth” and “3 months or more after birth”. The criteria to define this cutoff point were based on the literature, which shows that several factors can influence sexual function and the return to sexual activity immediately after childbirth, leading to reduced sexual interest or desire during this period ^18^ ^,^ ^19^ ^,^ ^20^.

### Complementary variables

The following covariates were used: (i) age, in years, of the pregnant woman (14-19, 20-24, 25 or more); (ii) skin color (white, black, mixed-race, yellow) ^30^; (iii) education of the pregnant woman, in years of study (0-8, 9-11, 12 or more); (iv) marital status of the pregnant woman (married, in a consensual union); (v) number of children in the household (no children, 1, 2 or more); (vi) occupation of the pregnant woman (no job, manual workers, non-manual workers); (vii) economic classification using the Brazilian Economic Classification Criteria ^31^ (A/B, C, D/E); (viii) partner’s age, in years (16-19, 20-24, 25 or more); (ix) partner’s education, in years of study (0-8, 9-11, 12 or more); (x) pregnancy planning (yes, no); and (xi) type of delivery (vaginal childbirth, cesarean section).

Continuous variables of the pregnant woman’s mental health were also assessed, such as level of perceived stress using the *Perceived Stress Scale* (PSS-14) ^32^; anxiety symptoms using the *Beck Anxiety Inventory* (BAI) ^33^; and symptoms of depression using the *Center for Epidemiologic Studies-Depression* scale (CES-D) ^34^. Social support was determined using the *Medical Outcomes Study* scale (MOS), translated into and adapted for Portuguese ^35^, with the results categorized as appropriate and inappropriate, using the upper tertile as the cutoff point ^36^.

### Data analysis

**Figure 2:**
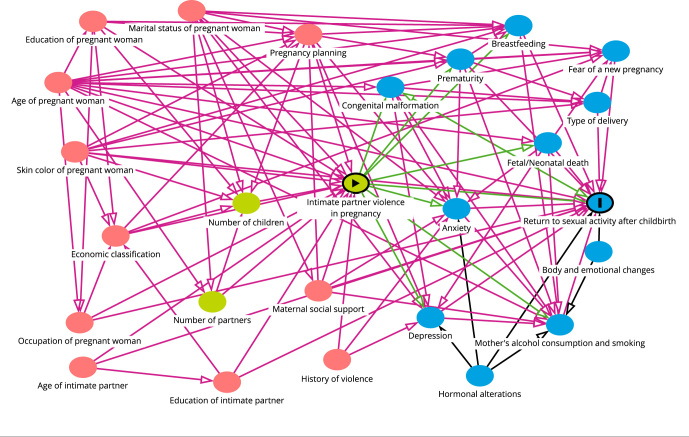
Directed acyclic graph for the association between intimate partner violence during pregnancy and time to return to sexual activity after childbirth. BRISA cohort, São Luís, Maranhão State, Brazil.

Source: prepared by the authors, using the DAGitty 3.0 software (http://www.dagitty.net/).

First, distributions of continuous variables were evaluated using the Shapiro-Wilk test for descriptive analyses. Absolute and relative frequency of categorical variables, median, and interquartile range of continuous variables were calculated. According to time to return to sexual activity after childbirth, categorical variables were compared using the chi-square test and continuous variables were compared using the Mann-Whitney test. A directed acyclic graph (DAG) was created in DAGitty 3.0 (http://www.dagitty.net/), which identified the minimum set of adjustment variables to minimize possible confounding biases, selecting the following variables: maternal social support, economic classification, skin color of pregnant woman, education of pregnant woman, education of intimate partner, history of violence, age of pregnant woman, age of intimate partner, occupation of pregnant woman, pregnancy planning, and marital status of pregnant woman (Figure 2). To assess the association between intimate partner violence during pregnancy and time to return to sexual activity after childbirth, logistic regression was used (crude and adjusted analysis) and the odds ratio (OR) was calculated with respective 95% confidence intervals (95%CI).

Statistical analysis of data was performed using Stata 14.0 program (https://www.stata.com).

### Ethical aspects

The study met the criteria of *Resolution n. 466/2012* of the Brazilian National Health Council. Every participant signed an informed consent form. The project was approved by the Research Ethics Committee of the HUMI/UFMA (opinion n. 223/2009, report n. 4771/2008-30).

## Results

The analytical sample of this sample consisted of 665 participants. Among them, 67.96% reported having returned to sexual activity within 3 months after childbirth. Most pregnant women were 25 years old or more (61.05%); self-declared mixed-race (66.92%), and had between 9 and 11 years of education (75.79%). Regarding marital status, 72.03% were in a consensual union, 54.89% did not live with children at the time of the interview, and 49.92% had no job. Regarding intimate partners, 74.14% of the participants reported their partners were 25 years old or more and 74.59% reported their partners had between 9 and 11 years of education. Most participants (69.32%) were from C socioeconomic group.

Regarding intimate partner violence during pregnancy, 24.06% of participants reported such violence. Regarding intimate partner violence 12 months prior to pregnancy, 25.26% reported it. Also, 74.89% of pregnant women reported inappropriate social support, 59.1% said they had not planned the pregnancy, and 52.03% of mothers reported vaginal delivery.

A significant difference was observed in time to return to sexual activity after childbirth in relation to some variables such as partner’s education and type of delivery. Regarding the presence of stress, and symptoms of anxiety and depression, the participants presented median of 21 (17-26) points on the PSS-14 scale, 14 (8-23) points on the BAI scale, and 10 (6-18) points on the CES-D scale (Table 1).

**Table 1:** Characterization of general sample and prevalence of return to sexual activity after childbirth according to demographic and socioeconomic variables and health conditions of participants in the BRISA cohort. São Luís, Maranhão State, Brazil, 2010-2011 (n = 665).

Characteristics	General sample (n = 665)	Return to sexual activity after childbirth		p-value *
		Within 3 months	More than 3 months	
	n (%)	n (%)	n (%)	
Age of pregnant woman (years)				0.92
14-19	65 (9.77)	43 (66.15)	22 (33.85)	
20-24	194 (29.17)	131 (67.53)	63 (32.47)	
25 or more	406 (61.05)	278 (68.47)	128 (31.57)	
Skin color of pregnant woman				0.49
White	113 (16.99)	78 (69.03)	35 (30.97)	
Black	97 (14.59)	66 (68.04)	31 (31.96)	
Mixed-race	445 (66.92)	299 (67.19)	146 (32.81)	
Yellow	10 (1.50)	9 (90.00)	1 (10.00)	
Education of pregnant woman (years of study)				0.25
0-8	76 (11.43)	56 (73.68)	20 (26.32)	
9-11	504 (75.79)	334 (66.27)	170 (33.73)	
12 or more	85 (12.78)	62 (72.94)	23 (27.03)	
Marital status of pregnant woman				0.30
Married	186 (27.97)	132 (70.97)	54 (29.03)	
Consensual union	479 (72.03)	320 (66.81)	159 (33.19)	
Number of child in the household				0.46
No child	365 (54.89)	241 (66.03)	124 (33.97)	
1	211 (31.73)	147 (69.67)	64 (30.33)	
2 or more	89 (13.38)	64 (71.91)	25 (28.09)	
Occupation of pregnant woman				0.06
No job	332 (49.92)	239 (71.99)	93 (28.01)	
Manual workers	210 (31.58)	131 (62.38)	79 (37.62)	
Non-manual workers	123 (18.50)	82 (66.67)	41 (33.33)	
Partner’s age (years)				0.93
16-19	14 (2.11)	10 (71.43)	4 (28.57)	
20-24	158 (23.76)	106 (67.09)	52 (32.91)	
25 old or more	493 (74.14)	336 (68.15)	157 (31.85)	
Partner’s education (years of study)				0.01
0-8	122 (18.35)	91 (74.59)	31 (25.41)	
9-11	496 (74.59)	322 (64.92)	174 (35.08)	
12 or more	47 (7.07)	39 (82.98)	8 (17.02)	
Economic classification				0.23
A/B	120 (18.05)	88 (73.33)	32 (26.67)	
C	461 (69.32)	304 (65.94)	157 (34.06)	
D/E	84 (12.63)	60 (71.43)	24 (28.57)	
Intimate partner violence during pregnancy (current or ex-partner)				0.53
No	505 (75.94)	340 (67.33)	165 (32.67)	
Yes	160 (24.06)	112 (70.00)	48 (30.00)	
History of violence (intimate partner violence 12 months before pregnancy)				0.47
No	497 (74.74)	344 (67.20)	163 (32.80)	
Yes	168 (25.26)	118 (70.24)	50 (29.76)	
Maternal social support				0.39
Appropriate	167 (25.11)	109 (65.27)	58 (34.73)	
Inappropriate	498 (74.89)	343 (68.88)	155 (31.12)	
Pregnancy planning				0.60
Yes	272 (40.90)	188 (69.12)	84 (30.88)	
No	393 (59.10)	264 (67.18)	129 (32.89)	
Type of delivery				< 0.01
Vaginal	346 (52.03)	255 (73.70)	91 (26.30)	
Cesarean	319 (47.97)	197 (61.76)	122 (38.24)	
	Median (IQR)	Median (IQR)	Median (IQR)	p-value **
PSS-14	21 (17-26)	21 (17-26)	21 (16-26)	0.86
BAI	14 (8-23)	15 (8-23)	13 (8-23)	0.40
CES-D	10 (6-18)	10 (6-17)	11 (6-18)	0.06

BAI: *Beck Anxiety Inventory*; CES-D: *Center for Epidemiologic Studies-Depression;* IQR: interquartile range; PSS: *Perceveid Stress Scale*.

* Chi-square test;

** Mann-Whitney test.

When analyzing the association between intimate partner violence during pregnancy and time to return to sexual activity after childbirth, no association was observed in the crude model (OR = 0.88; 95%CI: 0.60-1.30) or in the model adjusted for possible confounding variables (OR = 1.00; 95%CI: 0.61-1.63) (Table 2).

**Table 2:** Association between intimate partner violence during pregnancy and time to return to sexual activity after childbirth among participants in the BRISA cohort. São Luís, Maranhão State, Brazil, 2010-2011.

Intimate partner violence	Crude analysis		Adjusted analysis *	
	OR (95%CI)	p-value	OR (95%CI)	p-value
Did not suffer violence during pregnancy	1.00		1.00	
Suffered violence during pregnancy	0.88 (0.60-1.30)	0.53	1.00 (0.61-1.63)	0.99

95%CI: 95% confidence interval; OR: odds ratio.

* Analysis adjusted for: maternal social support, economic classification, skin color of pregnant woman, education of pregnant woman, education of intimate partner, history of violence, age of pregnant woman, age of intimate partner, occupation of pregnant woman, pregnancy planning, and marital status of pregnant woman.

As a sensitivity analysis, we assessed the association between each type of violence and time to return to sexual activity after childbirth (Table 3).

**Table 3:** Association between types of intimate partner violence during pregnancy and time to return to sexual activity after childbirth among participants in the BRISA cohort. São Luís, Maranhão State, Brazil, 2010-2011.

Types of violence	Crude model		Adjusted model *	
	OR (95%CI)	p-value	OR (95%CI)	p-value
Did not suffer violence **	Reference		Reference	
Psychological violence	0.82 (0.56-1.21)	0.32	0.91 (0.56-1.48)	0.70
Physical violence	0.74 (0.41-1.34)	0.31	0.82 (0.43-1.57)	0.58
Sexual violence	1.04 (0.59-1.81)	0.90	1.22 (0.67-2.22)	0.52
Physical and sexual violence	0.76 (0.43-1.31)	0.32	0.85 (0.46-1.57)	0.60

95%CI: 95% confidence interval; OR: odds ratio.

* Analysis adjusted for: maternal social support, economic classification, skin color of pregnant woman, education of pregnant woman, education of intimate partner, history of violence, age of pregnant woman, age of intimate partner, occupation of pregnant woman, pregnancy planning, and marital status of pregnant woman;

** Not have suffered violence relating to each specific type: psychological violence, physical violence, sexual violence, and physical and sexual violence.

## Discussion

The findings of this study did not show any association between intimate partner violence during pregnancy and time to return to sexual activity within 3 months after childbirth and more than 3 months after childbirth.

Although intimate partner violence against women can be considered a global problem, existing evidence is still not sufficient to explain different issues resulting from this phenomenon that negatively affect maternal and child health.

According to a literature review, only one study was found evaluating the relationship between intimate partner violence and sexual issues after childbirth related to depression. This review assessed 700 women treated in basic health units located in the west zone of São Paulo and did not show any association between studied exposure and outcome ^37^, in agreement with the results found in our study.

A common aspect between the two studies is the fact that they both assessed violence during pregnancy, ignoring violence that occurred after childbirth, a situation that may have influenced the results, as recent violence has a stronger impact on women’s health ^38^ and, consequently, may affect the return to sexual activity after childbirth.

Data about violence were collected only in the second trimester of pregnancy; therefore, the results reflect responses to questions about episodes of violence that occurred at two specific moments (before and during pregnancy). However, although violence in the postpartum period was not assessed in our study, it is important to consider that suffering violence during a delicate period such as pregnancy can be a predictor of future violence. A longitudinal study conducted with 1,083 women in Hong Kong to investigate the trajectory of partner violence before, during, and after pregnancy observed that a high proportion of women suffered intimate partner violence continuously during pregnancy and after childbirth ^39^.

In our study, the prevalence of women who suffered intimate partner violence during pregnancy was lower (24.06%) when compared to women who did not suffer intimate partner violence (75.94%). This result may suggest an underestimation of cases. According to the literature, several factors may obstruct the production of data on violence and pregnancy, including feeling of guilt, shame, fear and stigma that women who are victims of violence experience ^40^, as well as inadequate or late access to prenatal care, which may occur due to prohibition by the partner or deep psychological stress experienced by the pregnant woman ^41^.

Most women (67.96%) in our study reported return to sexual activity within 3 months after childbirth. Physical, psycho-emotional, and sociocultural aspects can inhibit desire, arousal, and lubrication, influencing the return to sexual activity in the postpartum period. Around half of women return to sexual activity between 5 and 6 weeks after childbirth, and around 90% are already sexually active 3 months after childbirth. However, when they do not return to sexual activity more than 12 weeks after childbirth, the situation must be evaluated, which may indicate a worse sexual prognosis and sexual inactivity ^15^ ^,^ ^42^.

Although it was not the primary objective of this study, a significant difference was found in time to return to sexual activity after childbirth in relation to some variables, such as type of delivery and partner’s education. Women who returned to sexual activity within 3 months had a higher prevalence of vaginal birth, while those who returned after 3 months had a higher prevalence of cesarean section. However, a recent review study showed that there is no consensus on an association between the type of delivery and changes in sexual function, showing that vaginal deliveries, whether instrumented or not, and cesarean deliveries, whether elective or emergency, can alter sexual function in the short, medium, and long term ^43^.

Regarding partner’s education, study participants who returned to sexual activity within 3 months stated that their partners had between 9 and 11 years of education (64.92%). No direct relationship was found in the literature between the partner’s education years and sexual activity in the postpartum period. Despite this fact, education is believed to influence other aspects of the relationship, such as communication and understanding of the importance of postpartum recovery. A population-based study found a statistically significant association between impaired communication and intimate partner violence. In this context, poor education can impact interpersonal relationships and the resolution of everyday problems, which can result in violence ^44^ ^,^ ^45^.

Also, a high prevalence of inappropriate social support among study participants should be highlighted, although this variable did not affect the time to return to sexual activity after childbirth.

Some studies report a relationship between sexual violence and female sexual dysfunction. This form of violence seems to have a more significant impact on a woman’s ability to maintain a satisfactory sexual life ^46^ ^,^ ^47^ ^,^ ^48^. However, in the sensitivity analysis conducted in our study, no statistically significant difference was observed between the type of violence and time to return to sexual activity after childbirth. Also, a low prevalence of women reporting sexual violence was observed in our study.

According to the literature, intimate partner violence is considered a stressor for many women and significantly contributes to mental disorders in the pregnancy-puerperal period, including depression, suicidal ideation, post-traumatic stress disorder, and anxiety disorders ^49^. Combined with that, studies indicate that psychological factors are involved in sexual behavior, desire, and satisfaction, in addition to postpartum depression, related to reduced frequency and interest in sexual relations between 8 and 12 weeks after birth and low sexual drive 6 months after childbirth when compared to women without postpartum depression ^50^. The result of non-association between intimate partner violence during pregnancy and time to return to sexual activity after childbirth found in our study may be related to non-inclusion of women’s mental health variables in the analysis, a situation justified by the fact that these variables appear as mediators, requiring another type of analysis not included in the objective of this study.

In our study, data on violence were based on the self-report of participants and their willingness to report true information, and women who are victims of partner violence often find it difficult to identify such violence. However, a self-administered instrument was used to reduce the chances of participant omission of violence and the identity of aggressors ^51^.

This instrument proved to be appropriate for the assessment of intimate partner violence against women, and it was used in other studies that also investigated the prevalence of intimate partner violence during pregnancy ^9^ ^,^ ^52^ ^,^ ^53^.

A limitation to be considered is the use of a convenience sample from the municipality of São Luís only, without representation of single women, since all participants included in the sample reported being married or in a consensual union. This lack of diversity may restrict the generalization of findings to a broader population.

Although this study did not present results about a possible association between intimate partner violence during pregnancy and time to return to sexual activity after childbirth, it is important to highlight methodological aspects regarding this analysis. Strengths include the fact that it is a cohort study; the use of instruments recognized and validated in Brazil, including the WHO VAW; and the use of a DAG to minimize possible confounding biases.

Also, to the best of our knowledge, no study was found in the literature assessing the same exposure and outcome, and many of the studies that address violence during pregnancy and sexuality in the postpartum period use a qualitative approach, which makes our study relevant.

## Conclusion

Although no association was found between intimate partner violence during pregnancy and time to return to sexual activity after childbirth, our study fills gaps in scientific knowledge about the negative outcomes of violence, suggesting new perspectives and paths for expanding comprehensive care to women’s health. Therefore, the importance of health professionals should be highlighted in terms of enforcement of policies to fight against violence and provision of continuous care to women in the pregnancy-puerperal cycle, considering physical and psychological aspects, as violence leads to painful consequences and negatively affects several aspects of women’s lives, including sexual and reproductive health.
